# *DGAT1* mutations leading to delayed chronic diarrhoea: a case report

**DOI:** 10.1186/s12881-020-01164-1

**Published:** 2020-12-01

**Authors:** Luojia Xu, Weizhong Gu, Youyou Luo, Jingan Lou, Jie Chen

**Affiliations:** 1 Present Address: Department of Gastroenterology, Children’s Hospital, Zhejiang University School of Medicine, National Clinical Research Center for Child Health, No.3333, Binsheng Road, Hangzhou, Zhejiang Province PR China; 2 Present Address: Department of Pathology, Children’s Hospital, Zhejiang University School of Medicine, National Clinical Research Center for Child Health, No.3333, Binsheng Road, Hangzhou, Zhejiang Province PR China

**Keywords:** Infantile diarrhoea, Failure to thrive, Genetics, Diacylglycerol o-acyltransferase, Case report

## Abstract

**Background:**

Early-onset chronic diarrhoea often indicates a congenital disorder. Mutation in *diacylglycerol o-acyltransferase 1* (*DGAT1*) has recently been linked to early-onset chronic diarrhoea. To date, only a few cases of *DGAT1* deficiency have been reported. Diarrhoea in those cases was severe and developed in the neonatal period or within 2 months after birth.

**Case presentation:**

Here, we report a female patient with *DGAT1* mutations with delayed-onset chronic diarrhoea. The patient had vomiting, hypoalbuminemia, hypertriglyceridemia, and failure to thrive at early infancy. Her intractable chronic diarrhoea occurred until she was 8 months of age. A compound heterozygous *DGAT1* mutation was found in the patient, which was first found in the Chinese population. Her symptoms and nutrition status improved after nutritional therapy, including a fat restriction diet.

**Conclusions:**

This case expanded our knowledge of the clinical features of patients with *DGAT1* mutations. Intractable diarrhoea with delayed onset could also be a congenital disorder.

**Supplementary Information:**

The online version contains supplementary material available at 10.1186/s12881-020-01164-1.

## Background

Chronic diarrhoea is defined as diarrhoea lasting longer than 2 weeks [1]. The aetiology of chronic diarrhoea varies [1]. A group of chronic diarrhoea disorders may occur in early infancy and can be termed “congenital diarrhoea” [1]. Many patients with disorders in this group are affected by monogenic disorders that primarily affect the intestinal epithelium or secondarily affect intestinal epithelial function [1]. With the increasing use of next-generation sequencing in clinical practice, many monogenic disorders that cause chronic diarrhoea have been revealed. One of these monogenic disorders is caused by a mutation in the *diacylglycerol o-acyltransferase 1* (*DGAT1*) gene (MIM #: 604900). The *DGAT1* gene encodes diacylglycerol o-acyltransferase 1 (DGAT1), which is a microsomal enzyme with high expression in the small intestine, adrenal cortex, adrenal medulla, and testes [2]. DGAT1 and its isozyme DGAT2 catalyse the final step in triglyceride (TG) synthesis by using diacylglycerol (DAG) and fatty acyl CoA in humans [3] and help to absorb TGs in the small intestine [2]. However, in humans, only *DGAT1* is highly expressed in the intestine [4].

Recent studies have shown that *DGAT1* mutation is linked to chronic and severe diarrhoea that mostly develops in the neonatal period [4–7] or within 2 months after birth [7, 8]. Herein, we report a female patient who had delayed-onset chronic diarrhoea with a compound heterozygous DGAT1 mutation, which, to our knowledge, has not been previously reported.

## Case presentation

The patient was a girl born at 41 weeks gestation with spontaneous labour. Her birth weight was 3.3 kg, and her length was 51.0 cm. Her mother denied any prenatal events. She came from a non-consanguineous Chinese family, and she was the first child without any siblings. She presented vomiting soon after birth but with normal stool output. The physical examination showed dehydration, but the rest of the examination was unremarkable. Her serum albumin and TG were in the normal range when she was 22 days old. She presented recurrent hypoalbuminemia and hypertriglyceridemia with a serum TG level of 8.34 mmol/L (normal range is < 1.70 mmol/L) and normal cholesterol since she was 34 days old. She began to have several episodes of watery diarrhoea daily when she was 8 months old. She was breastfed after birth and then changed to an amino acid-based formula or extensively hydrolysed-based formula because of a suspected cow’s milk protein allergy. Her routine stool test was normal, with negative results for viruses, bacteria, or parasites. In other laboratory results, immunoglobins decreased (IgG 0.96 g/L, IgA 0.15 g/L, IgM 0.32 g/L), and abnormalities were also revealed in the T and B cell subset analysis (CD20 14.22%, CD3 65.44%, CD4 20.56%, CD8 34.90%, NK cell 6.55%, CD4/CD8 0.59). Prealbumin and IgE levels were unremarkable most of the time. Her iron level was 5.51 mmol/L (normal range 6.63–11.82 mmol/L), which was slightly low, but zinc, magnesium, and copper levels were all within a normal range. Retinol-binding protein was lower at 19.40 mg/L (normal range: 22–53 mg/L), while vitamin D, vitamin B12, and folic acid levels were normal. Insulin-like growth factor-1 (IGF-1) was < 25.0 ng/ml (normal range 51.0–303.0 ng/ml), and insulin-like growth factor binding protein-3 (IGFBP-3) was 0.7 μg/ml (normal range 0.8–3.9 μg/ml). The upper gastrointestinal contrast study showed nothing but a gastroesophageal reflex. Oesophagogastroduodenoscopy with biopsy was performed, which showed slightly flat villi in the descending duodenum, and pathology results demonstrated slight inflammation at the gastric antrum mucosa and chronic mucosal inflammation in the descending part of the duodenum. A percutaneous endoscopic gastrostomy and a percutaneous endoscopic transgastric jejunostomy were performed during the whole treatment course. She was given either an amino acid-based formula or extensively hydrolysed-based formula using gastrostomy tube feeding or jejunal tube feeding. Because of poor feeding with ongoing loss from the gastrointestinal tract, parenteral nutrition was added. She was on parenteral nutrition for approximately 11 months with an interruption for catheter-related infection, thrombosis, or discharge against doctor’s orders. She had several episodes of bacterial or viral infections. Her parenteral nutrition provided a total energy of 71.4–153.5 kcal/kg daily with an amino acid supply of 2.5–3.5 g/kg daily, and she could tolerate a maximum dose of 3.8 g/kg/d lipid emulsion (including 2.0 g/kg/d medium-chain triglyceride/long-chain triglyceride fat emulsion and 1.8 g/kg/d ω-3 fish-oil lipid emulsion) without TG elevation or parenteral nutrition-associated cholestasis. She also received albumin, intravenous immune globulin infusion, and red blood cell transfusion when moderate anaemia occurred. When her feeding volume was decreased or she was put on nothing by mouth, her symptoms improved temporarily, and vice versa. Even then, she still suffered from a failure to thrive during hospitalization. Weight-for-age z-score (WAZ), length/height-for-age z-score (HAZ) and weight-for-length z-score (WHZ) were calculated by WHO Anthro software (version 3.2.2). Her WAZ and WHZ were always below -3. She was accidentally fed with rice porridge for a couple of days, and her symptoms were relieved when she was 16 months old. After her parents provided the consents, the whole-exome sequencing (WES) of peripheral blood from the proband was performed, and the Sanger sequencing was performed with her parents’ peripheral blood to confirm whether they had the same mutations as the proband.

**Fig. 1:**
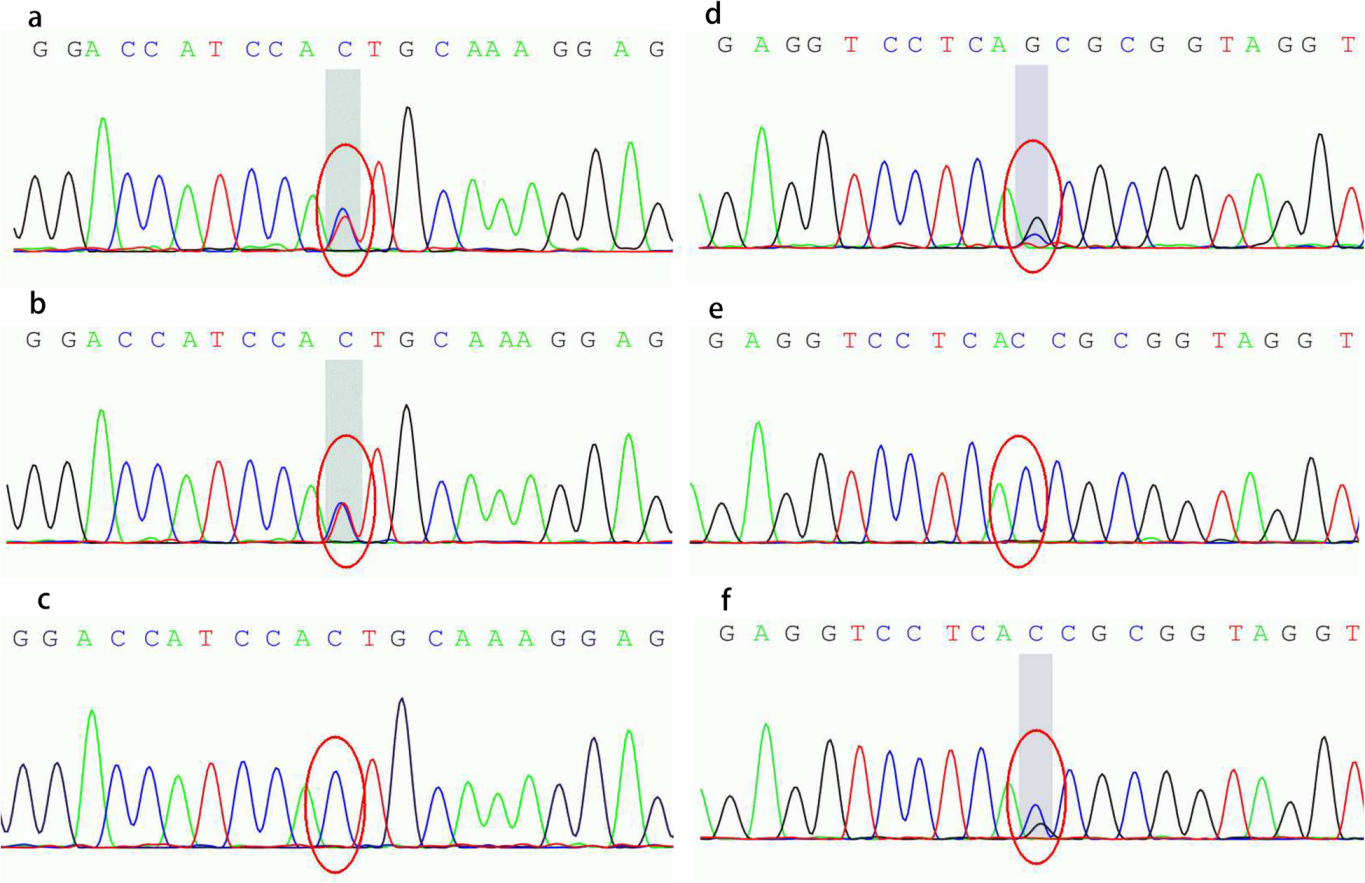
Sanger sequencing results show mutations in *DGAT1* detected in the proband’s family. **a** Chromatogram showing the proband with a heterozygous mutation at chr8:145541376, c.895-1G > A. **b** Chromatogram showing the proband’s father carrying the same heterozygous mutation, c.895-1G > A. **c** Chromatogram showing the proband’s mother with no mutation at this site. **d** Chromatogram showing the proband with another heterozygous mutation at chr8:145541757, c.751 + 1G > C. **e** Chromatogram showing the proband’s father with no mutation at this site. **f** Chromatogram showing the proband’s mother carrying the same heterozygous mutation, c.751 + 1G > C

WES was performed as described previously [9]. The analysis results showed that our patient had compound heterozygous mutations in *DGAT1*: a paternally inherited splicing mutation at chr8: 145541376 c.895-1G > A and a maternally inherited splicing mutation at chr8: 145541757 c.751 + 1G > C. The Sanger sequencing results are shown in Fig. 1. Her father and mother were heterozygous for each mutation.

The two mutations were both splicing mutations and could affect the results of transcription and translation. The analysis from the gnomAD database (gnomad.broadinstitute.org) showed that the mutation at chr8:145541376, c.895-1G > A was only reported in non-Finnish European populations with 1/251026 in total allele counts [10]. The other mutation was not previously reported in the gnomAD database. Use of GERP++ [11] suggested that the mutations were conserved. In addition, use of MutationTaster (http://www.mutationtaster.org) [12] to test the potential pathogenicity of these mutations revealed that they were disease-causing. Human Splicing Finder (http://www.umd.be/HSF/) [13] showed that these two mutations most likely affected splicing.

**Fig. 2:**
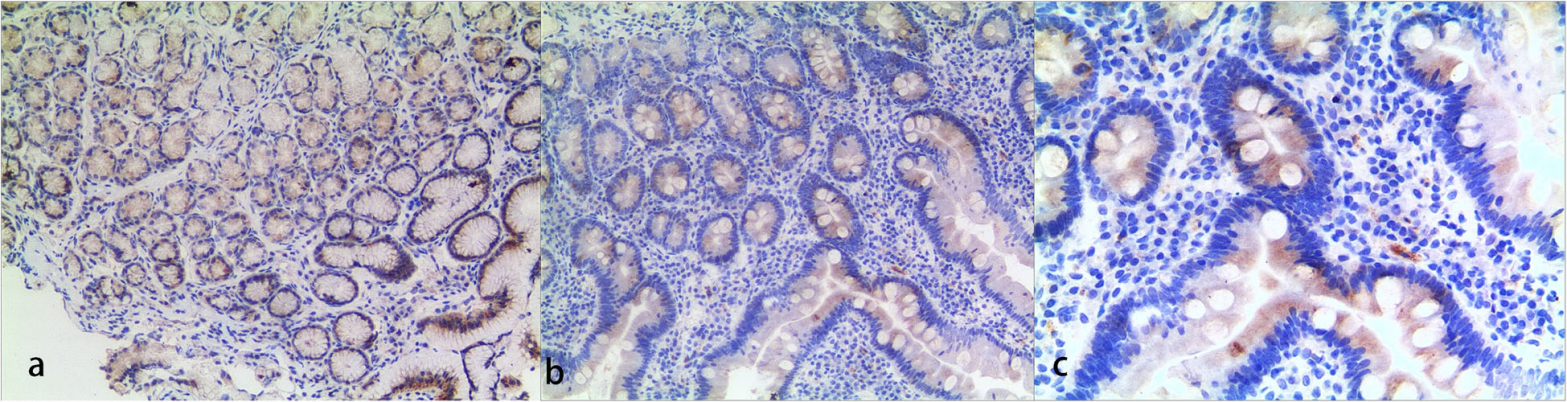
DGAT1 protein expression in the proband. **a** Immunohistochemistry of DGAT1 in the proband’s biopsy of the gastric antrum (× 100). **b** Immunohistochemistry of DGAT1 in the proband’s biopsy at the duodenum (× 100). **c** Immunohistochemistry of DGAT1 in the proband’s biopsy at the duodenum (× 200)

DGAT1 protein expression in enterocytes was assessed by immunohistochemistry (IHC) staining in the biopsy tissue. IHC processing followed the instructions and is described in Additional file 1. DGAT1 protein expression in the gastric antrum (Fig. 2a) and duodenum (Fig. 2b and c) of the proband was shown in Fig. 2. Additional figures of the IHC results in the duodenum can be seen in Additional file 2.

**Fig. 3:**
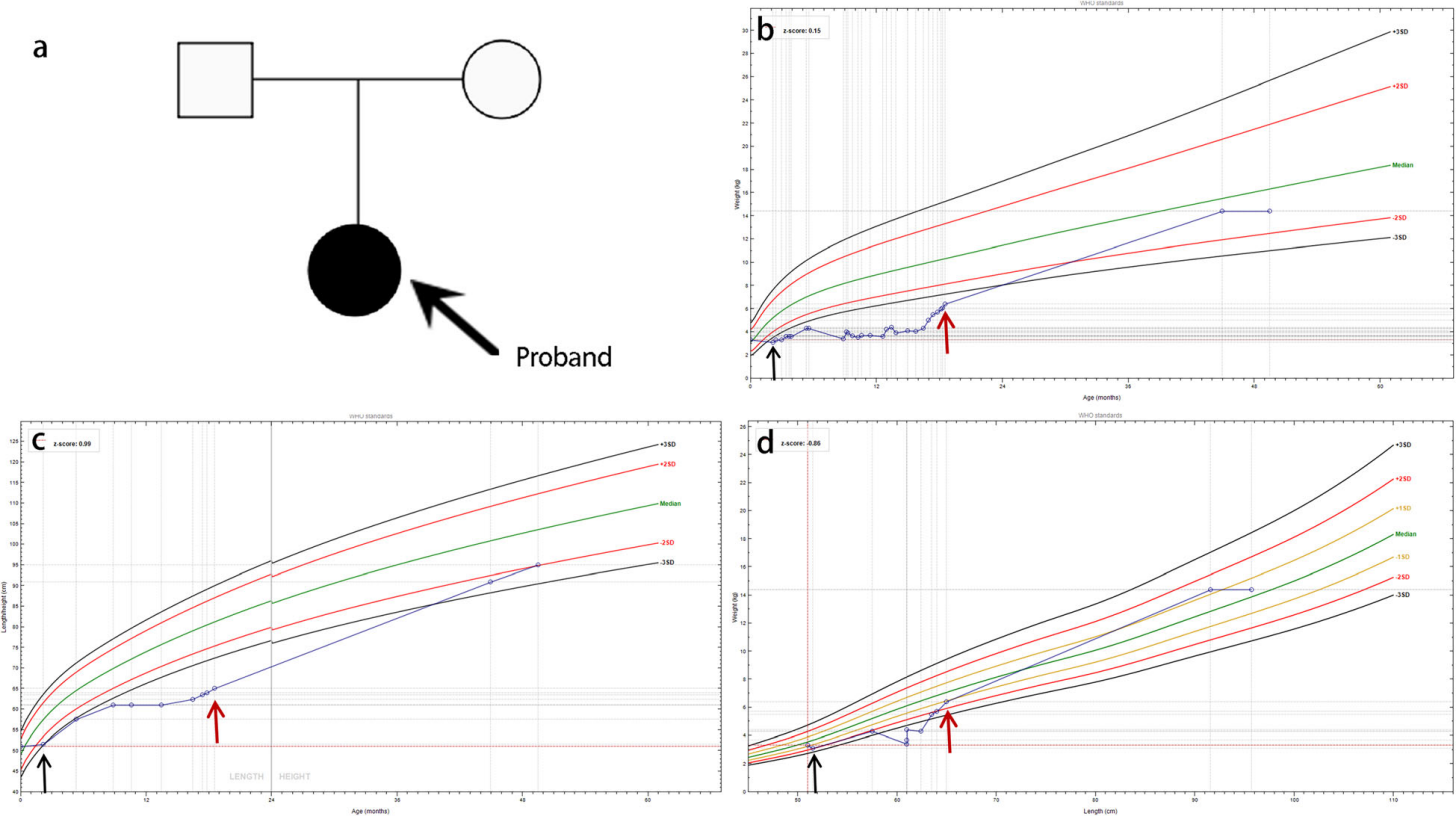
Pedigree of the proband’s family and the growth curve of the proband. **a** Pedigree of the affected family. The proband is indicated with a black arrow; it could be an autosomal recessive disease. **b** WAZ of the proband’s growth parameters. **c** HAZ of the proband’s growth parameters. **d** WHZ of the proband’s growth parameters. Short black arrows indicate the time when the proband received parenteral nutrition, and the red arrows indicate the time point when the proband started a fat-restricted diet

The proband was confirmed with a diagnosis of DGAT1 deficiency. As a result, she moved to a fat-restricted diet. She started with rice porridge and rice cereal, which were mainly carbohydrates. Lean meat and vegetables were introduced later as complementary feeding of semisolid foods. She did well without diarrhoea or vomiting. Finally, she was weaned off parenteral nutrition. She was 4 years and 2 months old at the last visit in the outpatient clinic, and she had an improved nutrition status (body weight 14.4 kg, height 95.0 cm, WAZ -0.92, WHZ 0.40, HAZ -1.97) with no signs of symptom recurrence. The pedigree of the proband’s family was shown in Fig. 3a. The growth curve was shown in Fig. 3b, Fig. 3c, and Fig. 3d. Albumin and immunoglobins remained in the normal range without the requirement of extra infusion. She had a normal TG level under a fat-restricted diet. IGF-1 and IGF BP-3 also returned to the normal range. Her parents were satisfied with this result.

## Discussion and conclusions

Fat is the main energy source for infants, and lipid metabolism widely exists in the human body, not only in different systems but also in different kinds of cells. DGAT1 plays an important role in TG synthesis [3]. Thus, theoretically, mutations in *DGAT1* can cause variant symptoms not only related to lipid absorption but also related to dysfunction of multiple organs. Human intestines may be more sensitive to *DGAT1* mutations because of the lack of expression of *DGAT2* [4]. Recent studies [4–8, 14] (Table 1) showed mutations in *DGAT1* from a total of 23 patients are linked to PLE with failure to thrive. These studies revealed that *DGAT1* mutations can lead to early-onset, chronic and intractable diarrhoea, which may develop into intestinal failure. In contrast with previously reported cases, our patient had diarrhoea until 8 months after birth despite her hypertriglyceridemia occurring in early infancy. This might have contributed to her compound heterozygous mutations in *DGAT1*. Another important symptom of patients with *DGAT1* mutations is vomiting. Our patient also had vomiting, which was similar to the reported 12 patients [4, 7, 8]. A previous study [15] may explain the potential reason: intestinal *DGAT1* deficiency inhibits chylomicron secretion and delays gastric emptying. Other clinical characteristics that our patient had, such as failure to thrive, hypoalbuminemia, and decreased serum immunoglobins, have also described in previous studies [4–8].

**Table 1:** Characteristics of published DGAT1 deficiency patients

Patient	Descent	Onset age	Features	Nutrition support	Other Treatment	Outcome
Patient 1 [4]	Ashkenazi Jewish	0.1 month	vomiting, diarrhoea, malnutrition, hypertriglyceridemia, hypoalbuminemia	TPN, gastrostomy tube feeding with amino acid-based formula	albumin infusion	died
Patient 2 [4]	Ashkenazi Jewish	0.1 month	diarrhoea, hypertriglyceridemia, hypoalbuminemia	TPN and amino acid-based formula feeding	albumin infusion, cholestyramin	thrived at 46 months of age on an unrestricted diet
Patient 3 [5]	Arab-Muslim	2 months	diarrhoea, extremity edema, cachectic with FTT, hypertriglyceridemia, hypoalbuminemia	amino acid-based formula feeding, later with PN and Monogen formula	albumin and IVIG infusion, metronidazole infusion	discharged home without diarrhoea
Patient 4 [5]	Arab-Muslim	NM	diarrhoea, edema, weight loss and skin abscesses	NM	NM	died
Patient 5 [5]	Ashkenazi Jewish	0.27 months	diarrhoea, FTT, edema, hypotonia	TPN and amino acid-based formula feeding	chicken soup formula mixed with rice water and ORS	improved on a regular diet except for dairy products
Patient 6 [5]	Ashkenazi Jewish	sooner after birth	diarrhoea, hypertriglyceridemia	TPN	chicken soup formula mixed with rice water and ORS	maintained his weight albumin on enteral feeding
Patient 7 [5]	NM	NM	NM	NM	NM	NM
Patient 8 [5]	NM	NM	NM	NM	NM	NM
Patient 9 [6]	South Asian	shortly after birth	diarrhoea, FTT, hypertriglyceridemia	PN, gastrostomy tube feeding with a hydrolyzed formula	blood cell transfusion, IVIG	transitioned to a diet containing no more than 10% calories from fat
Patient 10 [6]	South Asian	shortly after birth	diarrhoea	NM	NM	improved on a very low fat enteral diet.
Patient 11 [7]	Turkish	birth	vomiting, diarrhoea, FTT, hypoalbuminemia	Fat-free fomula and MCT	albumin infusion	no diarrhoea and normal growth with low-fat diet +MCT + fat-free formula
Patient 12 [7]	Turkish	birth	vomiting, diarrhoea, FTT, hypoalbuminemia	NM	albumin infusion	died
Patient 13 [7]	Turkish	0.75 months	vomiting, diarrhoea, FTT, hypertriglyceridemia, hypoalbuminemia	TPN	albumin infusion	diarrhoea improved with basic formula feeding
Patient 14 [7]	Turkish	2 months	vomiting, diarrhoea, FTT, hypoalbuminemia	NM	cholestyramine	diarrhoea improved
Patient 15 [7]	Turkish	1.3 months	vomiting, diarrhoea, FTT, hypoalbuminemia	hydrolyzed formula	Creon pancreatic lipase	diarrhoea improved but remained malnutrition
Patient 16 [7]	Turkish	2.5 months	vomiting, diarrhoea, FTT	NM	Creon pancreatic lipase	diarrhoea improved
Patient 17 [7]	Caucasian	1 month	vomiting	infusion of Intralipid and Omegaven suppletion of lipid soluble vitamins	NM	enteral feeding without fat
Patient 18 [7]	Caucasian	1 month	vomiting, FTT	infusion of Intralipid and Omegaven suppletion of lipid soluble vitamins	NM	enteral feeding without fat
Patient 19 [7]	Caucasian	birth	vomiting, diarrhoea, FTT, hypoalbuminemia	TPN	small bowel transplantation	still stunted
Patient 20 [7]	Caucasian	birth	vomiting, diarrhoea, FTT, hypoalbuminemia	TPN, fat-free formula	NM	still stunted
Patient 21 [8]	Caucasian	1 month	vomiting, diarrhoea, malnutrition, hypoalbuminemia	TPN, nasojejunal tube feeding with amino acid-based formula	antibiotics	improved on a low-fat diet
Patient 22 [14]	Chinese	birth	vomiting, watery diarrhoea	TPN	albumin and IVIG infusion	died
Patient 23 [14]	Chinese	birth	edema,watery diarrhoea	TPN, enteral nutrition	albumin and IVIG infusion	died

*TPN* Total parenteral nutrition, *PN* Parenteral nutrition, *IVIG* Intravenous immune globulin, *NM* Not mentioned, *FTT* Failure to thrive, *MCT* Medium-chain triglyceride, *ORS* Oral rehydration solution

Mutations in *DGAT1* leading to protein expression deletion or downregulation are still controversial. One study [7] revealed that some *DGAT1* mutations can cause a lack of DGAT1 protein expression in the human epithelial cells of the duodenum, ileum, colon, patient-derived fibroblasts, Epstein-Barr virus-derived B-lymphoblastic cell lines and patient-derived intestinal organoids. In addition, another two studies [5, 6] revealed that some mutations in *DGAT1* can cause partial function loss, which leads to decreased expression in patients’ dermal fibroblasts. The differences may be due to the different locations of the mutation in the *DGAT1* gene. Further functional analysis of mutated alleles is needed to obtain a better understanding of the genotype-phenotype relationships in *DGAT1* mutation patients.

The *DGAT1* mutations in our patient were two compound mutational sites in the *DGAT1* exon region, which were located in splice sites and probably affected the function of the protein. They were likely pathogenic. The patient’s clinical characteristics and immunohistochemical results of the duodenum were also in line with the pathogenicity of such compound heterozygous mutations. To date, several homozygous mutations have already been identified in Turkish Caucasians in the Netherlands and South Asian, Ashkenazi Jew, Arab-Muslim, and Chinese populations [4–7, 14]. There is one case of compound heterozygous mutations in Caucasians [8]. This is the first described compound heterozygous mutation in the Chinese population.

How the variant mutations in *DGAT1* are linked to such critical disease remains unknown. One [7] of those studies explored the possible molecular pathomechanism: lipotoxicity in the intestinal epithelium accompanied by *DGAT1* deficiency leads to mucosal injury, which developed clinical features of PLE. However, to date, those reported patients had variant onset ages of different symptoms. Further investigation will be needed to explore the deeper mechanism. Autophagy or endoplasmic reticulum stress is also possibly involved in this process and has already been implicated in other lipid metabolic disorders [16].

For the early diagnosis of this disease, patients usually show signs of PLE with failure to thrive in early infancy, but delayed intractable diarrhoea is also one of the clinical characteristics. The faecal elastase level is not a specific indicator [7] but can be used to rule out some other diseases with similar symptoms. The faecal alpha-1-antitrypsin level is helpful to establish the diagnosis of PLE [17]. Although *DGAT1* mutations affect lipid metabolism, not all patients have hypertriglyceridemia [5–7]. Including our patient, 7 in 24 patients [5–8] were reported to have hypertriglyceridemia. Unexplained hypertriglyceridemia in early life may be a strong indicator of lipid metabolic disorder. Patients always have a failure to thrive with extremely poor nutrition status, but prealbumin may be at a normal level as in our patient. In PLE patients, albumin synthesis increases while enteric albumin is lost [18], and the body cannot compensate completely [19]. Thus, proteins with longer half-lives, such as albumin and immunoglobulins, are affected, but proteins with shorter half-lives, such as prealbumin, are less affected. In our patient, IGF-1 and IGFBP-3 both decreased in the early clinical course and reversed to normal levels during the follow-up when her nutrition status improved. This indicates that IGF-1 and IGFBP-3 change secondary to malnutrition, which is consistent with some previous findings [20, 21]. Most of the patients [4, 5, 7], including our patient, have recurrent infections. This may be due to decreased immunoglobin levels secondary to malnutrition and long-term use of central lines.

Nutrition therapy with a fat-restricted diet is an effective way to manage these patients. When reviewing diet changes in 16 surviving patients, 9 patients showed a good response to a low-fat diet (Table 1). One of them transitioned to a regular diet, and the remaining 8 patients were still on a low-fat diet [6–8]. One study [6] reported that the patient had a diet in which fat supplied less than 10% calories. Our patient showed great improvement after a fat-restricted diet was introduced. Since an analysis [22] says that approximately 95–98% of breast milk fat is TG, breastfeeding may not be a good choice for babies with *DGAT1* deficiency. The patient reported here also showed intolerance of breastfeeding. Parenteral nutrition plays an important role even when the exact aetiology is unclear. When the genetic test and immunohistochemical results confirm the diagnosis, the transition to a fat-restricted diet will still need parental nutrition to provide essential fatty acids and fat-soluble vitamins. Thus, occasional infusion of lipid emulsion with fat-soluble vitamins is necessary [7]. In our experience, early intervention with parenteral nutrition prevents fat-soluble vitamin deficiency despite the unclear aetiology. Our patient tolerated a maximum dose of 3.8 g/kg/d lipid emulsion without lipid clearance problems. Parental nutrition lasting for 11 months was safe, but body weight gain remained poor, which might be due to the DGAT1 deficiency mediating lipid metabolic disorders. Fat-soluble vitamins, such as vitamin A and vitamin D, were maintained in the normal range even when parenteral nutrition stopped as she was discharged. Quality of life and prevention of infections should also be the physician’s concerns.

In summary, we reported a patient with *DGAT1* compound heterozygous mutations in the Chinese population who presented delayed chronic diarrhoea. This case expanded our knowledge of the clinical features of patients with *DGAT1* mutations. Paediatricians should be alert to this disease when infants have delayed intractable diarrhoea with failure to thrive. We had only one case here, and more studies focusing on the mechanism of lipid metabolism in DGAT1-deficient patients will be necessary in the future.

## Supplementary Information

**Additional file 1.** Description of immunohistochemical analysis.

**Additional file 2.** Immunohistochemistry of DGAT1 in the duodenum.

## Data Availability

WES data of the proband were deposited in the NCBI Sequence Read Archive (SRA) database (Project ID: PRJNA673384). All datasets generated or analysed during the current study are included in this published article and available from the corresponding author on reasonable request. Database analysed in the study included gnomAD (gnomad.broadinstitute.org).

## Abbreviations

DGAT1: Diacylglycerol o-acyltransferase 1
TG: Triglyceride
DAG: Diacylglycerol
PLE: Protein-losing enteropathy
IHC: Immunohistochemistry
IGF-1: Insulin-like growth factor-1
IGF BP-3: Insulin-like growth factor binding protein-3
WAZ: Weight-for-age z-score
HAZ: Length/height-for-age z-score
WHZ: Weight-for-length z-score
WES: Whole-exome sequencing
