# Supplementary material for: DGAT1 mutations leading to delayed chronic diarrhoea: a case report
Source: BMC Med Genet. 2020 Dec 1;21:239. doi: 10.1186/s12881-020-01164-1 (PMC7708908; doi:10.1186/s12881-020-01164-1)
Supplement: Supplementary file 1 — Additional file 1. Description of immunohistochemical analysis. [file 12881_2020_1164_MOESM1_ESM.docx]

Description of immunohistochemical analysis

The immunohistochemistry(IHC) analysis was done following the DAKO EnVision^TM^ kit instructions. The specimens were dewaxed, incubated with methanol containing 30% H_2_O_2_ for 20 minuets, blocking endogenous peroxidase activity, then immersed in 0.01 mol/L citrate buffer (pH 6.0), heated at 100°C in a microwave oven for 20 minuets, washed 3 times with distilled water and blocked with 1% BSA for 30 min. The specimens were incubated overnight at 4°C with rabbit polyclonal IgG of Anti-DGAT1 antibody (Abcam, ab54037) at a 1:250 dilution. Then use second antibodies (Dakocytomation Company, Denmark) at 37°C for 30 minuets. specimens were washed 3 times with phosphate-buffered saline (PBS) and subsequently the color was displayed with DAB (Dakocytomation Company, Denmark) for about 5 minuets.
