# Supplementary material for: DGAT1 mutations leading to delayed chronic diarrhoea: a case report
Source: BMC Med Genet. 2020 Dec 1;21:239. doi: 10.1186/s12881-020-01164-1 (PMC7708908; doi:10.1186/s12881-020-01164-1)
Supplement: Supplementary file 2 — Additional file 2. Immunohistochemistry of DGAT1 in the duodenum. [file 12881_2020_1164_MOESM2_ESM.pptx]

## Slide 1
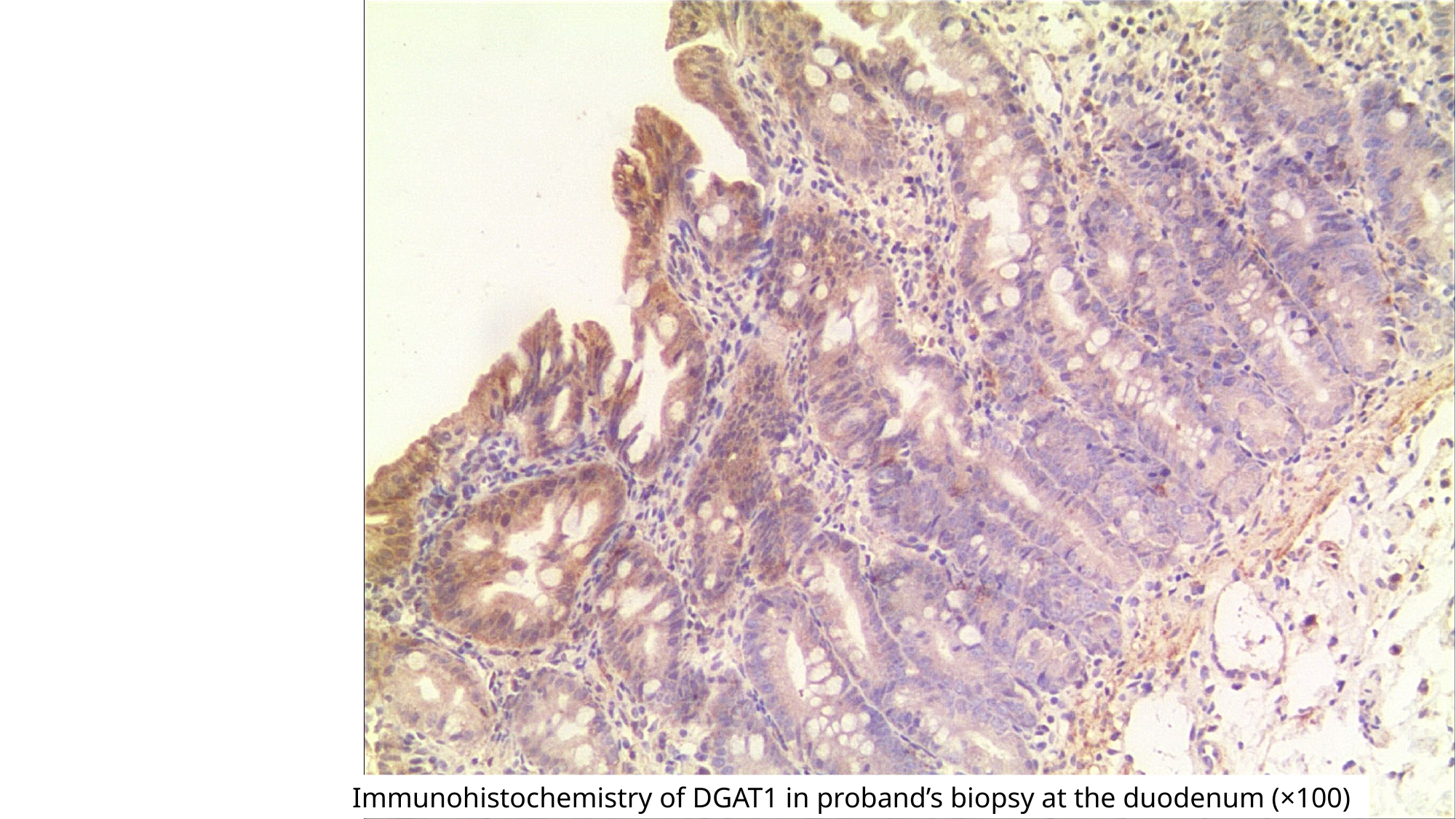

Immunohistochemistry of DGAT1 in proband’s biopsy at the duodenum (×100)

## Slide 2
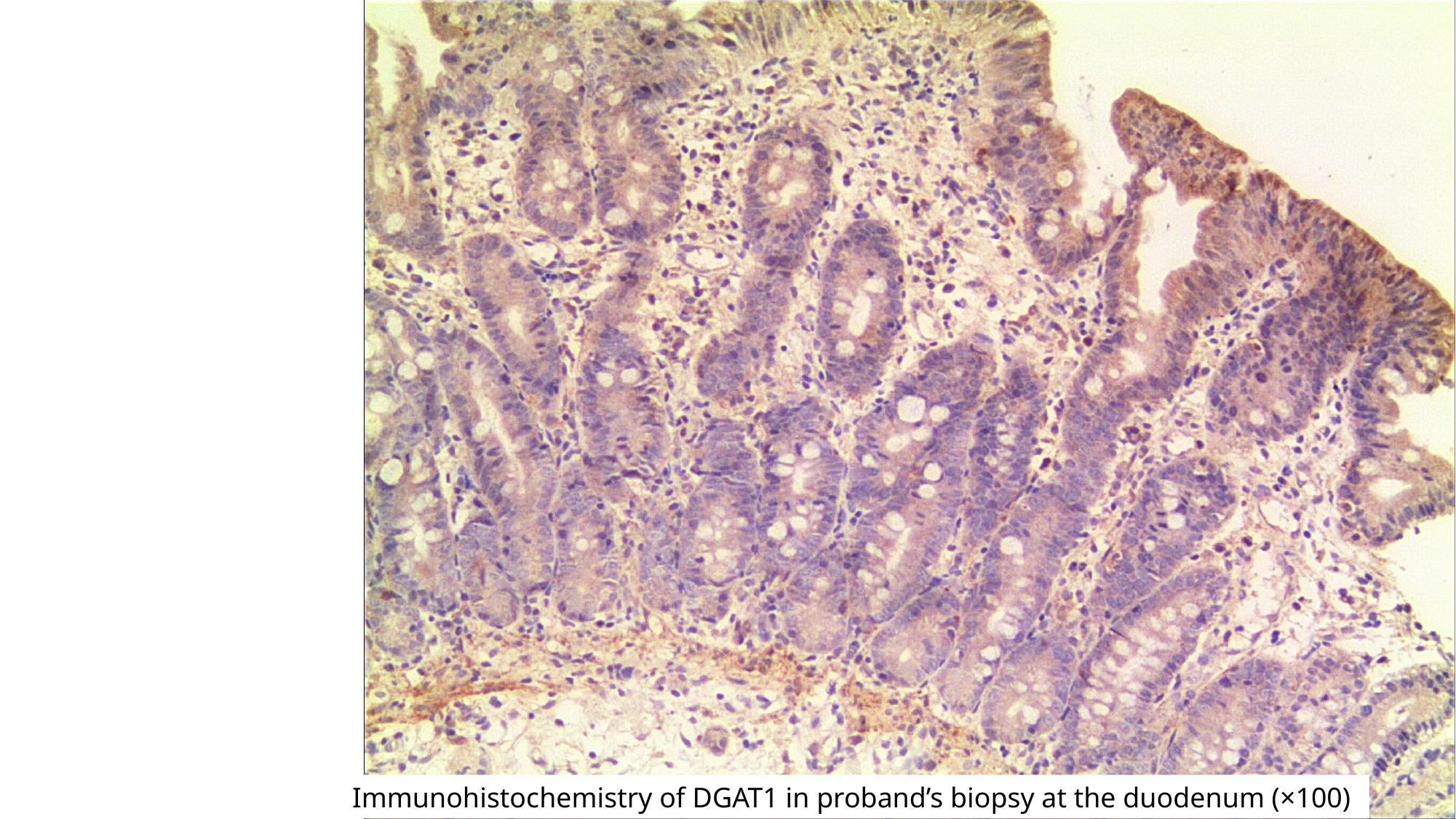

Immunohistochemistry of DGAT1 in proband’s biopsy at the duodenum (×100)

## Slide 3
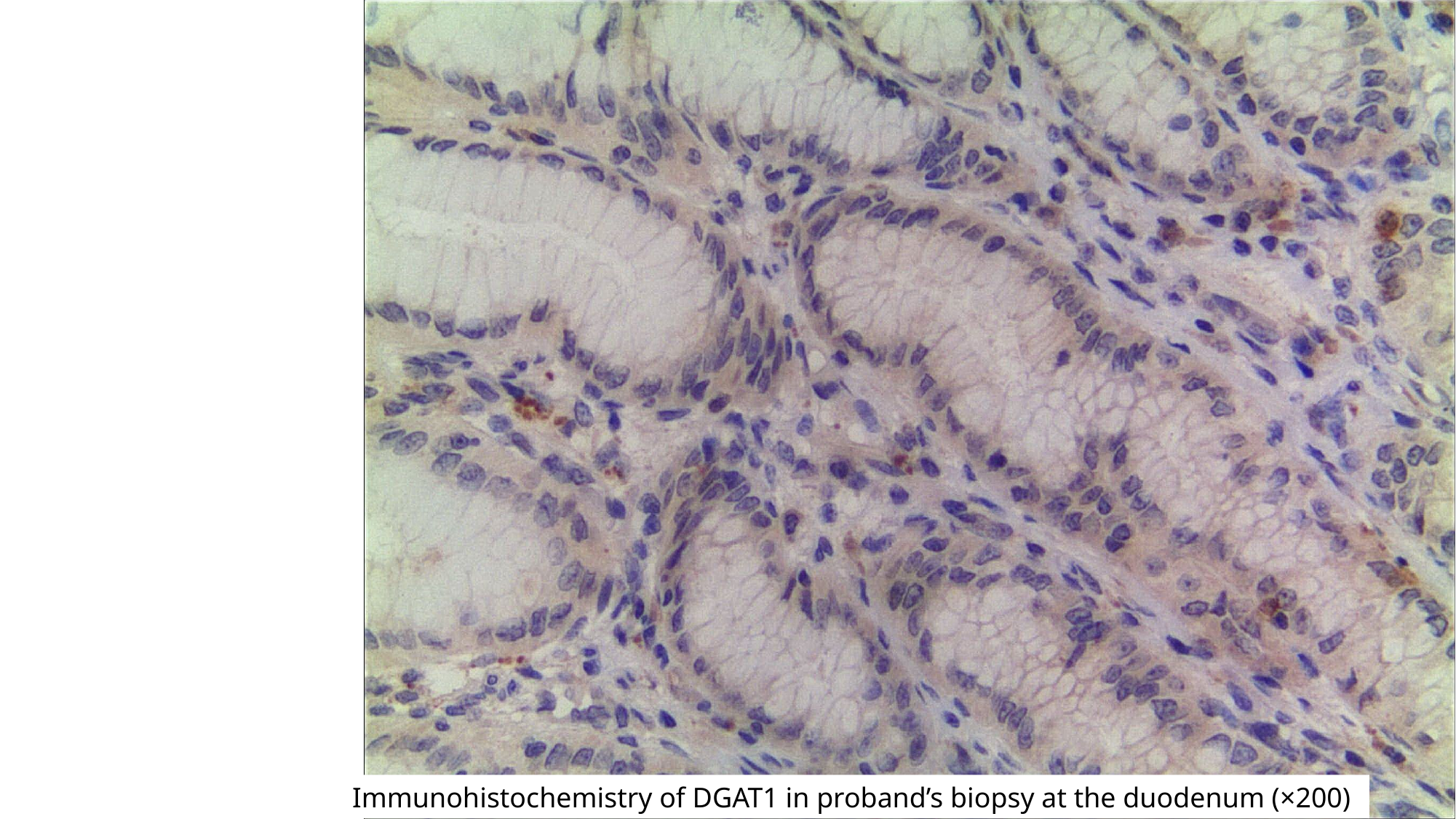

Immunohistochemistry of DGAT1 in proband’s biopsy at the duodenum (×200)

## Slide 4
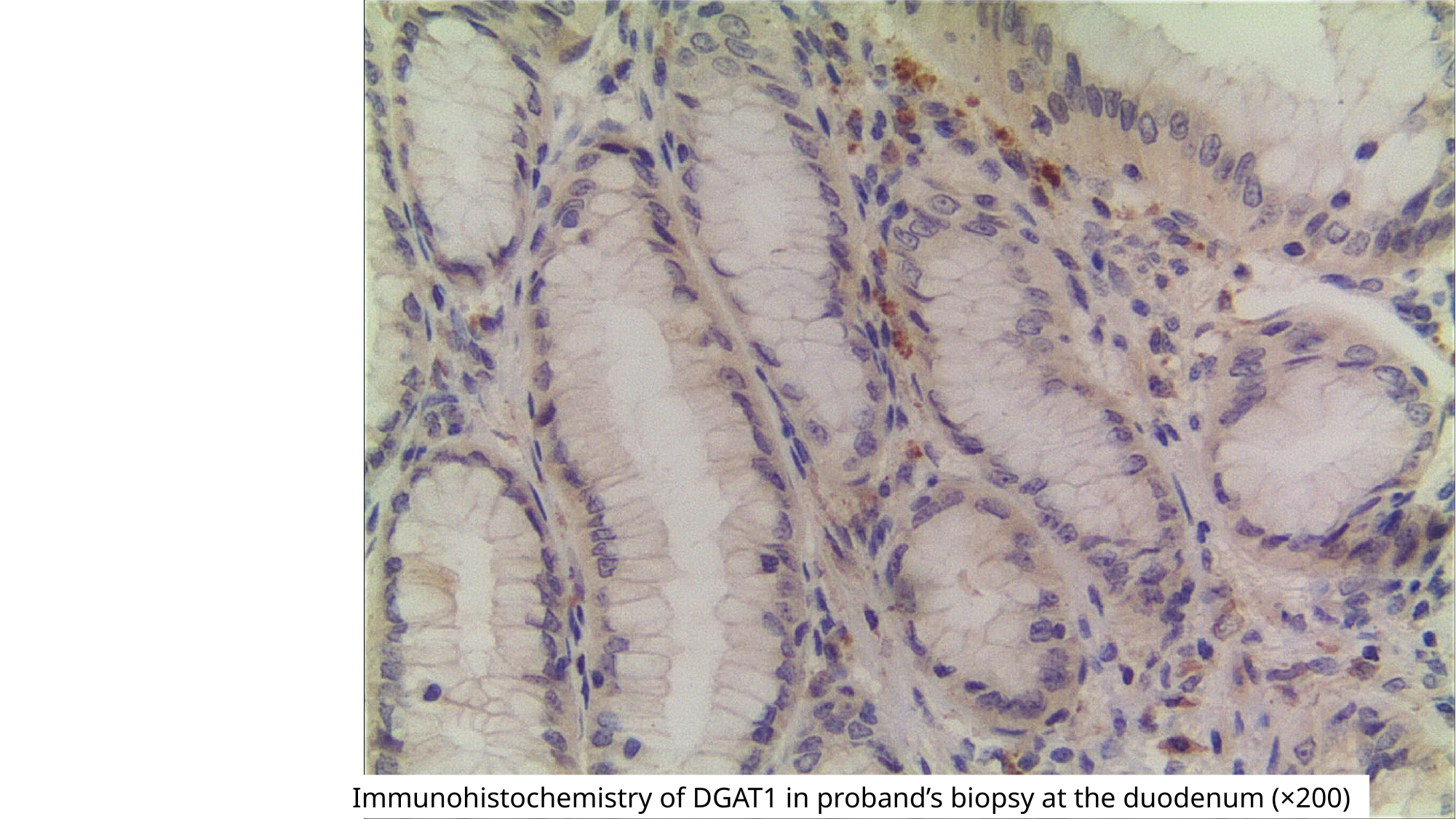

Immunohistochemistry of DGAT1 in proband’s biopsy at the duodenum (×200)
